# Supplementary material for: Rural Family Caregiving: A Closer Look at the Impacts of Health, Care Work, Financial Distress, and Social Loneliness on Anxiety
Source: Healthcare (Basel). 2022 Jun 21;10(7):1155. doi: 10.3390/healthcare10071155 (PMC9318565; doi:10.3390/healthcare10071155)
Supplement: Supplementary file 1 [file healthcare-10-01155-s001.zip › healthcare-1737881-supplementary.pdf]

healthcare

File S1: **Semi-structured interview guide**

Family Caregiver Interview Guide

Overview:

The purpose of the interview today is to hear from you how providing care has changed during the pandemic, what you think could have been done to support you and the person that you care for during the pandemic, and then what you think you will need as the COVID-19 is controlled.

Guiding questions:

1. First, can you tell us a little bit about your caregiving situation, as much or as little as you feel comfortable with.
   Probes:
   - How has your caregiving changed during the COVID-19 pandemic?
   - What has been your biggest worry during the COVID-19 pandemic?
2. What might have made your life/ caregiving easier during the pandemic?

   Probes
   - What resources you have benefitted from?
   - What do you think should have been done differently?
3. What could have been done to support you and the person you care for during the pandemic

   Probes:
   - Has anyone asked you about your wellbeing and your caregiving?
   - Have you experienced any relief or positive experiences during the COVID-19 pandemic?
   - Did you experience any difficulties in coordinating care because of COVID-19 restrictions?
4. Now, as the pandemic seems to be controllable and things become more normal, what do you think you need?

   Probes:
   - What resources might help you to care?
   - What might make it easier for you to care?
   - What might help you to maintain your own health?

Concluding question:

5. Now I have asked you lots of questions, what do you think we should know about?

Conclusion:

Thank you for participating. Your experiences and recommendations are important for us to help make policymakers and the public aware of what family caregivers do and what they need. If you think of anything else that you would like to tell us, you have my email address and cell phone number. Thank you again

COVID-19 has Affected Alberta Family Caregivers. What is happening in 2021?

16 months into the COVID19 pandemic, Dr. Jasneet Parmar and her research team from the University of Alberta are inviting you to participate in a survey to understand how Alberta Family Caregivers are doing right now in the summer of 2021 and what family caregivers need to thrive going forward.Principal Investigator: Dr. Jasneet Parmar Phone: 780-901-6236 jasneet.parmar@albertahealthservices.ca

Affiliations: Department of Family Medicine, Faculty of Medicine and Dentistry, University of Alberta, (Edmonton, Alberta)

Funding: Northern Alberta Academic Family Medicine Fund

The COVID-19 pandemic has had a profound effect on all people in Canada, including family caregivers in Alberta. We define family caregiver (carer, care partner) as any person who takes on a generally unpaid caring role providing emotional, physical or practical support in response to another person's illness, disability or age-related needs.

Why are you being asked to take part in this survey? You are a family caregiver who lives in Alberta or a caregiver of someone who lives in Alberta.

Study Purpose. We hope to understand the impact of the COVID-19 pandemic and public health physical distancing measures on you, your workload, how you have managed and adapted, and your needs going forward. By sharing your opinions and experiences, you will help Dr. Jasneet Parmar and her research team at the University of Alberta plan better supports for family caregivers during this COVID-19 pandemic and beyond.

How many people will participate in the survey? According to Statistics Canada data, there are almost a million family caregivers in Alberta. However, we do not know how many Alberta family caregivers will participate in the survey given their busy lives, but we estimate it could range between 100 and 1000 people.

What will happen if I participate in this survey? We are inviting family caregivers from Alberta to complete this voluntary survey. The survey should take you approximately 15 to 20 minutes depending on how you respond to questions. Your responses will remain anonymous. No personal or identifying information is being collected and all data will be analyzed as a group (aggregated). At the end of the survey, you will be asked whether you would like to be contacted for a follow-up interview about the impact of COVID-19 on you in your caregiving role. You can participate in this survey regardless of whether you choose to be part of the follow-up study. You will also have the option to join the draw and win one of two $50 gift certificates! Participation in the draw is optional.

What are the potential risks or discomforts? Some questions that ask about how the COVID-19 physical distancing has affected you or the person you care for may make you feel personally uncomfortable. Please note that you do not have to answer any questions you do not wish to answer or make you feel uncomfortable.

What are the potential benefits? There are no direct benefits to survey participants. However, the results of the survey may influence services to better support family caregivers. Results will be shared with family caregivers, Caregivers Alberta, and the Alberta Caregiver Focused Coalition to strengthen their programs and assist with advocacy.

Do I have to participate? Completing the survey is your choice. You have the option to not answer questions if you do not want to and still continue the survey. You can exit the survey at any time by closing the survey. Because we are not collecting personal identifying information, we cannot remove the answers once you have completed the survey.

Will my information be kept confidential? Your responses to this survey are completely confidential. It will not be possible to identify you in any report. All data will be kept confidential and stored securely. We are using a secure online platform called REDCAP. Researcher access to the survey Is password-protected and transmission is encrypted. Survey responses cannot be linked to your data.

How long will the information be kept? In accordance with the University of Alberta data retention policy, we will keep the data for five years after completion of the study (2026).

If you have questions or concerns about this study, please contact Dr Parmar or Dr. Sharon Anderson the research coordinator sdanders@ualberta.ca .

Questions and Concerns:

If you have any questions about this survey, or if you would like us to mail to you a paper copy of the study, please contact Dr. Sharon Anderson at 780-953- 5541 or sdanders@ualberta.ca

This study has received approval by the University of Alberta Health Ethics Research Board Study Number

PRO0097996. If you have questions regarding your rights as a study participant, please call the University of Alberta Research Ethics Office at 780-492-2615.

By continuing with the survey your consent to participate is implied.

1. Are you a family caregiver?<br>A family caregiver (carer, care partner) is any person who takes on a generally unpaid caring role providing emotional, physical, or practical support in response to another person's disability, mental illness, drug or alcohol dependency, chronic condition, dementia, terminal or serious illness, frailty from ageing, or COVID-19.	◯ Yes, I am caring for a family member, friend, or neighbor now.<br>◯ I have not provided this support the past 12 months<br>◯ No, I am not providing care.<br>◯ Don't know/ Not sure
2. Do you live in Alberta?	◯ Yes<br>◯ No
3. What are the first three digits of your postal code?	________________________

We have a few questions about the care you provide.

1. In an average week, how much time do you spend providing care? There are 168 hours in a week.

◯ 1 hour or less
◯ 2 to 9 hours
◯ 10 hours
◯ 11 to 20 hours
◯ 21 to 30 hours
◯ 31 to 40 hours
◯ 41 to 80 hours
◯ 81 to 120 hours
◯ 121 to 168 hours
◯ Prefer not to answer

2. Has the amount of care you provide to a family member, friend, or neighbor changed since the COVID-19 pandemic began (March 2020)?

◯ Yes, I am providing MORE care since the COVID- 19 pandemic began.
◯ No, I am providing the SAME AMOUNT of care as I did before the COVID-19 pandemic.
◯ Yes, I am providing LESS care than I was before the COVID-19 pandemic.
◯ Not applicable, I started caring since the COVID-19 pandemic began in March 2020.

2A How long have you been providing care? (please answer with full number of years e.g., 3, 6,, 10)

____

3. Which of the following types of care and support do you provide to another person due to their health condition or limitation? Please select all that apply

☐ Emotional and social support
☐ Managing care such as making appointments
☐ Shopping for or delivering food/supplies
☐ Help with home maintenance or outdoor work
☐ Transportation, including trips to the doctor, social visits, or for shopping
☐ Help with preparing food, housekeeping, laundry
☐ Handling finances
☐ Assistance with eating, dressing, managing medications
☐ Intimate care such as bathing, showering, toileting, managing incontinence
☐ Medical care such as help with nursing care (e.g., wound care/bandage changes, foot care managing medical equipment)
☐ other

3A You checked other to the question above. What other types of care and support do you provide to another person you care for?

____

4. Going forward after dealing with the COVID-19 pandemic for 16 months, how confident do you feel as a caregiver? Please select one answer.

◯ Not at all confident
◯ Somewhat confident
◯ Very confident
◯ Don't know/ Not Sure

5. After dealing with the COVID-19 pandemic for 16 months, please tell us about what you need going forward to support your caregiving and/or to maintain your own health and wellbeing.

____

Section 2: Demographics. Can you tell us about you ?

1. How old are you?

- ◯ Under 14
- ◯ 15 to 24
- ◯ 25 to 34
- ◯ 35 to 44
- ◯ 45 to 54
- ◯ 55 to 64
- ◯ 65 to 74
- ◯ 75 to 84
- ◯ 85 to 94
- ◯ 95 years of age and older

2. What is your gender? Which of the following do you identify with? Please select one answer.

- ◯ Woman
- ◯ Man
- ◯ Transgender
- ◯ Non-binary
- ◯ Other

3. What is the highest level of education that you have completed? Please select one answer.

- ◯ Elementary /Middle School or Junior High
- ◯ High school, general or vocational
- ◯ College (pre-university), technical training (NAIT/SAIT), certificate, accreditation or advanced diploma)
- ◯ Undergraduate degree or some university
- ◯ Post-graduate degree or professional designation

4. To which ethnicity or population group do you belong? Please select all that apply

- ☐ Aboriginal / First Nations
- ☐ Arabic (Middle East, North Africa)
- ☐ Black (African, African-American, etc.)
- ☐ Caucasian (white)
- ☐ Chinese
- ☐ Filipino
- ☐ Japanese
- ☐ Korean
- ☐ Latin American (Mexican, Chilean, Costa Rican, etc.)
- ☐ South Asian (Indian, Bangladeshi, Pakistani, Sri Lankan, etc.)
- ☐ Southeast Asian (Vietnamese, Cambodian, Malaysian, etc.)
- ☐ West Asian (Iranian, Afghan, etc.)
- ☐ Other

5. What is your current employment status? Please select one answer

- ☐ Employed - Full-time
- ☐ Employed - Part time
- ☐ Employed - Self employed
- ☐ High school student
- ☐ Post-secondary student
- ☐ Student (Other)
- ☐ Retired
- ☐ Homemaker
- ☐ Disabled/on disability supports
- ☐ Unemployed
- ☐ Laid off temporarily
- ☐ Other

6. Has your employment status changed as a result of caregiving? Please select all that apply

- ☐ Working fewer hours
- ☐ Working more hours
- ☐ Laid off temporarily
- ☐ Loss of employment
- ☐ Working from home
- ☐ On a leave of absence
- ☐ No impact
- ☐ Other

7. Is there anything you would like to tell us about the impact of caregiving on your employment during the pandemic?

____

Section 3 This section is about how caregiving and COVID-19 have affected your finances.

1. During the pandemic, have you experienced financial hardship because of your caregiving responsibilities? Please select one answer.

- ◯ No financial hardships because of caregiving responsibilities.
- ◯ A few financial hardships because of my caregiving responsibilities.
- ◯ Moderate financial hardships because of my caregiving responsibilities.
- ◯ Yes, a lot of financial hardships because of my caregiving responsibilities.

2. Which of the following Federal benefits (including COVID-19 specific benefits) have you made use of? Please select all that apply

- ☐ EI Caregiving Benefits and Leave (including Compassionate Care Benefit (CCB)
- ☐ Employment Insurance (EI) Family Caregiver Benefit (max 15 weeks)
- ☐ Employment Insurance (EI)Compassionate Care Benefit (CCB)
- ☐ Canada Caregiver Credit (CCC)
- ☐ Canada Recovery Caregiving Benefit (CRCB)
- ☐ Canada Recovery Sickness Benefit (CRSB
- ☐ Canada Recovery Benefit (CRB)
- ☐ The Canadian Emergency Response Benefit (CERB)
- ☐ The Canadian Emergency Wage Subsidy (CEWS)
- ☐ Child disability benefit
- ☐ Canada Pension Plan (CPP) Disability Benefit
- ☐ CPP children's benefits for dependents under 25 benefits?
- ☐ Caregiver Recognition Benefit for veterans
- ☐ Other
- ☐ None
- ☐ Don't know

3. Which of the following items, if any, did you increase your spending on during the COVID-19 pandemic because of your caregiving responsibilities? Please select all that apply

- ☐ Food
- ☐ Care supplies (e.g., incontinence products, bandages, Meal replacements such as Ensure)
- ☐ Medical expenses (Rx Co-pay, over the counter medicines)
- ☐ Mobility equipment (co-pay, purchase) walkers, wheelchairs, scooters
- ☐ Household expenses (e.g., rent, mortgage, taxes, utility bills, insurance)
- ☐ Personal Items (e.g., clothing, shoes, pajamas)
- ☐ Protective Equipment/ PPE
- ☐ Technology (e.g., cell phones, computers, tablets, tech accessories)
- ☐ Other
- ☐ None of the above/ no extra spending

4. Which of the following private services did you pay for personally to support you in your role as a caregiver since the COVID-19 pandemic started? Please select all that apply	☐ Personal care such as assistance with eating,<br>☐ Dressing, grooming, bathing, or toileting<br>☐ Medical care such as help taking medicine or help with nursing care (for example, dressing changes or foot care)<br>☐ Managing care such as making appointments<br>☐ Help with activities such as housework, home maintenance or outdoor work<br>☐ Transportation, including trips to the doctor or for shopping<br>☐ Meal preparation or delivery<br>☐ Emotional support<br>☐ Other<br>☐ None, I did not pay for private services since the COVID-19 pandemic began
5. Going forward after 16 months of the COVID-19, please tell us, what kind of financial supports would assist you with your caregiving or to maintain your own health and wellbeing.	__________________________________

Section 4 Please help us to understand how you feel right now in July of 2021. We are going to ask about your health and wellbeing

1. Over the last year, my PHYSICAL health has	◯ Has improved<br>◯ Remained about the same<br>◯ Has deteriorated (e.g. less fit, more pain, new illness)
2. Over the past year, my MENTAL health has	◯ Has improved<br>◯ Remained about the same<br>◯ Has deteriorated (e.g. more worried, stressed, anxious, depressed)

3. COVID-19 may have affected your social relationships and networks. For each statement, please tell us how much you agree with the statements with Yes, More or Less, or No.

	Yes	More or Less	No
A. I often feel rejected	◯	◯	◯
B. I experience a general sense ◯ of emptiness	◯	◯	
C. I miss having people around ◯ me	◯	◯	
D. There are plenty of people I ◯ can rely on when I have problems	◯	◯	
E. There are many people I could trust completely	◯	◯	◯
F. There are enough people I ◯ feel close to	◯	◯	

4. COVID-19 may have affected stress and anxiety. Think about how you feel today. For each statement, please indicate how much you agree with the statement, Not at all, Somewhat, Moderately, Very Much.

	Not at all	Somewhat	Moderately	Very Much
A. I am comfortable	◯	◯	◯	◯
B. I am anguished	◯	◯	◯	◯
C. I feel at ease	◯	◯	◯	◯
D. I feel nervous	◯	◯	◯	◯
E. I feel concerned	◯	◯	◯	◯
F. I feel good	◯	◯	◯	◯

	Strongly Disagree	Disagree	Agree	Strongly Agree
5. In the past month, I was not able to take a break from caregiving.	◯	◯	◯	◯
6. In the past month, I couldn't ◯ stop thinking about all the caregiving activities I have to do.	◯	◯	◯	
7. In the past month, I felt ◯ frustrated due to my caregiving role.	◯	◯	◯	
8. In the past month, I did not ◯ sleep well due to my caregiving role.	◯	◯	◯	

9. We would like to know how YOU are managing overall. Which of the following statements describes you right now? Select one answer.

◯ I am active, energetic, and exercise regularly.
◯ I am well, but only occasionally active. I can manage finances/transportation/heavy housework on my own.
◯ My health conditions are well managed, but I am generally inactive. I may require advice on how to obtain supports with finances/transportation/heavy housework.
◯ I am more tired than I used to be, and have more troubles obtaining supports than before, but still can coordinate things myself.
◯ I need physical or practical assistance with finances, transportation, or heavy housework.
◯ I need assistance with out of home activities, struggle with stairs and could use help with my bathing or medications.
◯ I need help with all my personal care
◯ I am completely dependent for all personal care (dressing, eating, help to go to bathroom).
◯ I am very ill and near the end of my life.

10. How would you rate your ability to provide emotional support as a caregiver to your care recipient? Please select one answer.

◯ Not at all capable
◯ A little capable
◯ Neutral
◯ Somewhat capable
◯ Very capable
◯ Don't know

11. How would you rate your ability to access services and navigate the healthcare system? Please select one answer.

- ◯ Not at all capable
- ◯ A little capable
- ◯ Neutral
- ◯ Somewhat capable
- ◯ Very capable
- ◯ Don't know

12. Given your current health and caregiving role, do you feel you can continue caring for your family member or friend? Please select one answer.

- ◯ Yes definitely
- ◯ Yes somewhat
- ◯ No
- ◯ They no longer need care or support
- ◯ Don't know

Section 5 The following questions are about your experiences with the health care system--family doctors, healthcare providers, hospitals, homecare, supportive living, or long-term care.

1. In the last 6 months, have ANY health care professionals asked you about your needs as a family caregiver?

- ◯ Yes
- ◯ No
- ◯ Not applicable because I have not interacted with health providers
- ◯ Not sure

1A. If you answered yes, who were the healthcare professionals who asked you about your caregiving situation or how you were doing as a caregiver? Check all that apply?

- ☐ My Family Doctor
- ☐ The Family Doctor of the person I care for
- ☐ Healthcare provider in a primary care network or a family doctor's office
- ☐ Home care professional (e.g., case manager, rec therapist)
- ☐ Healthcare provider in a hospital
- ☐ Healthcare provider in supportive living
- ☐ Healthcare provider in long-term care
- ☐ Healthcare provider in private community practice (e.g, psychologist, social worker, physiotherapist)
- ☐ Other

2. Were you or the person you are caring for receiving Home Care services? Please select one answer.

- ☐ Yes
- ☐ No
- ☐ Pending - will be soon
- ☐ Other
- ☐ Don't know

2A. Have Home Care services changed for you or the person you are caring for since the COVID-19 pandemic started? Please select one answer

- ◯ MORE services because the person I care for has higher needs
- ◯ MORE services because the person I care for has long-lasting COVID-19
- ◯ SAME service as before COVID-19
- ◯ LESS service because of the COVID-19 pandemic
- ◯ LESS service because the person I care for has fewer needs
- ◯ LESS service because I discontinued it out of fear of contracting COVID-19
- ◯ Services have been suspended as a result of the COVID-19 pandemic
- ◯ Other
- ◯ Don't know

3. Now going forward, please tell us about what healthcare services would support your caregiving and help you to maintain your own health and wellbeing.

____

Section 6 Almost done, we need to ask you a few questions about the person or people that you care for.

1. How many people do you provide care for? Please enter a whole number

____

When answering the next set of questions about the person you care for, please think about the person you SPEND the MOST TIME caring for.
2. What is your relationship to the care receiver you spend the most time caring for? Please select one answer

- ◯ Parent/In-Law
- ◯ Spouse/Partner
- ◯ Sibling
- ◯ Child
- ◯ Other Relative
- ◯ Friend/Neighbor
- ◯ Employee
- ◯ Other

3. Where does person you SPEND THE MOST TIME CARING FOR live right now? Please select one answer.

- ◯ They live with you in the same household as you do.
- ◯ They live separately in their own private household (house, condo, apartment)
- ◯ They live in supportive living (e.g., lodge, assisted living)
- ◯ They live in long-term care or a group home
- ◯ Other

You answered that you care for someone in "other" living situation. Please tell us where they are living?

____

3A If the person you care for lives in a lodge, group home, supportive living, long-term care, or an auxiliary hospital, did you consider bringing the person you care for home because of the COVID- 19 pandemic?

- ◯ Yes
- ◯ No

4. How old is the person you SPEND THE MOST TIME CARING FOR.

____

5. What conditions, disabilities, or illnesses does the person you SPEND THE MOST TIME CARING FOR have? Check all that apply.

- ☐ Autism Spectrum Disorder
- ☐ Acquired Brain Injury, Stroke, Head injury
- ☐ Cancer
- ☐ Chronic health condition (e.g., Diabetes, arthritis, asthma, Crohn's, Cystic Fibrosis, COPD)
- ☐ COVID-19
- ☐ Dementia
- ☐ Drug or Alcohol dependency
- ☐ Frailty due to aging
- ☐ Heart Disease
- ☐ Intellectual disability
- ☐ Mental Illness Substance Use
- ☐ Neurological condition (e.g., epilepsy, Parkinson's Disease, Multiple Sclerosis)
- ☐ Sensory impairment (e.g., hearing loss, vision loss, blind)
- ☐ Terminal or serious illness
- ☐ Other
- ☐ Don't know

6. We would like to know how the person you spend the most time caring for is able to manage overall. Please select one answer that best fits with their situation and how much assistance they need to manage.

- ◯ They are active, energetic, and exercise regularly.
- ◯ They are well, but only occasionally active. They can manage finances/transportation/heavy housework on their own.
- ◯ Their health conditions are well managed, but they are generally inactive. They may require advice on how to obtain supports with finances/transportation/heavy housework.
- ◯ They need more help than they used to need, and have more troubles obtaining supports than before, but still can coordinate things by themselves.
- ◯ They need physical or practical assistance with finances, transportation, housework or yardwork.
- ◯ They need assistance with out of home activities, struggle with stairs, and need help with bathing or medications.
- ◯ They need help with all personal care (preparing meals, medications, showering,)
- ◯ They are completely dependent for all personal care tasks (dressing, eating, going to bathroom).
- ◯ They are very ill or frail, near the end of life, or palliative.

7. How long can the person you SPEND THE MOST TIME CARING FOR. be left alone?

- ◯ A week
- ◯ A few days but less than a week
- ◯ One day
- ◯ 4 or more hours but less than a day
- ◯ Less than 4 hours
- ◯ Not at all
- ◯ Don't know

8. During the COVID-19 pandemic so far, the health of the person you SPEND THE MOST TIME CARING FOR has

- ◯ Improved
- ◯ Remained the same
- ◯ Deteriorated (e.g., more depressed, worried, forgetful, or frail)
- ◯ Don't know

9. Is there anything else that you want to tell us about the health or care needs of the person(s) you care for?

____

We have asked you many questions, is there anything that you would like to tell us about caregiving or what you think needs to be done to support family caregivers who are caring for Albertans living in community homes, in supportive living, or long-term care ?

____

File S3: Clinical Frailty Scale- Self-report version

1. I am active, energetic, and exercise regularly.
2. I am well, but only occasionally active. I can manage finances, transportation, and heavy housework on my own.
3. My health conditions are well managed, but I am generally inactive. I may require advice on how to obtain supports with finances, transportation, or heavy housework
4. I am more tired than I used to be, and have more trouble obtaining supports than before, but can still coordinate things myself.
5. I need physical or practical assistance with finances, transportation, or heavy housework.
6. I need assistance with out of home activities, require help with bathing or medications, or struggle with stairs.
7. I need help with all of my personal care.
8. I am completely dependent for all of my personal care.
9. I am terminally ill and at the end of my life.

File S4: Stages of Thematic Analysis

Step 1	Researchers familiarize themselves with data	Read transcripts and listen to the recordings. Make corrections in transcriptions, Make notes on tone, first impressions of the data.
Step 2	Identify preliminary codes	Identify interesting elements in data. Document impressions with notes.<br>Write memos on emerging themes.<br>Document connected elements.
Step 3	Find themes in the data	Look for and identify themes in the data.<br>Identify all data relevant to the themes.
Step 4	Finalize the themes	Finalize the themes. Check for data overlaps between the themes.
Step 5	Review each theme	Review the data to ensure that data fits each unique theme. Reread the transcriptions to ensure the overall data fits with the overall storyline and each theme.
Sept 6	Document and review documented analysis.	Analyze the resultant documentation and the inferences drawn.

File S5: Types of support provided by caregivers in the community and in congregate care

Types of Support Provided				
Type of support	**Provided**	**Total Rural**	**Caregivers of Community-Dwelling Receivers**	**Caregivers of Congregate Care Residents**
Emotional and social support		126	82	38
	Yes	121(96%)	79 (96%)	37 (97%)
	No	5 (4%)	3 (4%)	1 (3%)
Managing care such as making appointments		126	82	38
	Yes	100 (79%)	72 (88%)	24(63%)
	No	29 (21%)	3 (12%)	14 (37%)
Shopping/delivering food/ supplies		126	82	38
	Yes	94 (75%)	65 (79%)	24 (63%)
	No	32 (25%)	17 (21%)	14(39%)
Home maintenance/ outdoor work		126	82	38
	Yes	61 (48%)	53 (65%)	5 (13%)
	No	65 (52%)	29 (35%)	33 87%)
Transportation. Trips to medical appts, social visits, shopping		126	82	38
	Yes	104 (83%)	70 (85%)	28(74%)
	No	22 (17%)	12 (15%)	10(26%)
Preparing Food, housekeeping laundry		126	82	38
	Yes	79 (63%)	64 (78%)	10(26%)
	No	47 (37%)	18 (22%)	28 (73%)
Handling Finances		126	82	38
	Yes	80 (64%)	52 (63%)	24 (63%)
	No	46 (36%)	30 (37%)	14 (37%)
Assistance with eating, dressing, managing medications		126	82	38
	Yes	60 (47%)	46 (56%)	10 (26%)
	No	66 (52%)	36 (44%)	28 (74%)
Intimate care bathing, toileting managing incontinence		126	82	38
	Yes	42 (33%)	35 (43%)	3(8%)
	No	84 (67%)	47 (57%)	36(92%)

File S6: Expenses that increased due to caregiving responsibilities.

Increased Expenses due to caregiving

Type of expense	Increased	Total Rural	Caregivers of Community-Dwelling Receivers	Caregivers of Congregate Care Residents	Chi Square *p*
Food	No	114	39 (34%)	75 (66%)	.042
	Yes	64 (56%)	27 (69%)	37 (49%)	
		50 (44%)	12 (31%)	38 (51%)	
Care supplies (incontinence products, bandages meal replacements)		114	39 (34%)	75 (66%)	.392
	No	82 (72%)	30 (77%)	52 (71%)	
	Yes	32 (28%)	9 (23%)	22 (29%)	
Medical Expenses (Rx Co-pay, over the counter medicines)		114	39 (34%)	75 (66%)	.049
	No	87 (76%)	34 (87%)	53 (71%)	
	Yes	27 (24%)	5 (13%)	22 (29%)	
Mobility equipment (Co-pay purchase walkers, wheelchairs, scooters		114	39 (34%)	75 (66%)	.087
	No	96 (84%)	36 (92%)	60 (80%)	
	Yes	18 (16%)	3 (8%)	15 (20%)	
Household expenses, e.g, rent, mortgage, taxes, utility bills insurance		114	39 (34%)	75 (66%)	.358
	No	91 (80%)	33 (85%)	58 (77%)	
	Yes	23 (20%)	6 (15%)	17 (23%)	
Personal items (e.g., clothing, shoes, pajamas)		114	39 (34%)	75 (66%)	.030
	No	89 (78%)	35 (90%)	54 (72%)	
	Yes	25 (22%)	4 (10%)	21 (28%)	
Personal Protective Equipment (e.g., Masks, gloves, hand sanitizer)		114	39 (34%)	75 (66%)	.004
	No	70 (61%)	31 (80%)	70 (61%)	
	Yes	44 (39%)	8 (20%)	44 (39%)	
Technology (cell phones, computers tablets, tech accessories)		114	39 (34%)	75 (66%)	.152
	No	81 (71%)	31 (80%)	50 (67%)	
	Yes	33 (29%)	8 (20%)	25 (33%)	

File S7: CHERRIES checklist: The Checklist for Reporting Results of Internet E-Surveys

Design	Describe survey design	Section 2 Paragraph 1
IRB (Institutional Review Board) approval and informed consent process.	IRB approval.	Section 2.3.2 University Health Ethics Research Board.
	Informed consent.	Section 2.3.2 Implied Consent
	Data protection.	Section 2.3.2 REDCap secure data collection platform.
Development and pre-testing.	Development and testing.	Section 2.4 Questionnaire reviewed by the research team and then Online REDCap survey reviewed by 5 family caregivers.
	Open survey versus closed survey.	Section 2.4 open survey
	Contact mode.	Section 2 .3.2
Recruitment process and description of the sample having access to the questionnaire.	Advertising the survey.	Section 2.3.2
	Web/Email.	Participants directed to REDCap link.
Survey administration.	Context.	Delivered on REDCAP
	Mandatory/voluntary.	As per ethics, only first 2 qualifying questions were mandatory.
	Incentives.	Draw for 2, $50 gift cards, Explanation included in Ethics Information about the Survey (Supplementary File 1).
	Time/Date.	Section 2.3.2 June 21 to August 31, 2020.
	Randomization of items.	No.
	Adaptive questioning.	Yes, Branching questions in REDCap. e.g. questions about homecare or care location only to those answering yes to those situations.
	Number of Items.	Sections were displayed as one page in REDCap.
	Number of screens (pages).	8 sections/ 8 pages.
	Completeness check.	Yes, Completed by REDCap platform and then manually
	Review step.	Yes participants could review/change answers and REDCap download PDF of responses was enabled.
Response rates.	Unique site visitor.	As per ethics anonymity we did not gather IP addresses or use cookies.

	View rate (Ratio of unique survey visitors/unique site visitors).	Not possible to calculate.
	Participation rate (Ratio of unique visitors who agreed to participate/unique first survey page visitors),	Section 3 Results 1st paragraph 58.9% (highlighted),
	Completion rate (Ratio of users who finished the survey/users who agreed to participate),	Section 3.2.1 81.2%
	Cookies used,	As per ethics anonymity we did not use cookies,
Preventing multiple entries from the same individual,	IP check,	As per ethics anonymity we did not use cookies,
	Log file analysis,	Checked for identical responses.
	Registration,	No,
Analysis,	Handling of incomplete questionnaires,	Those with less than 80% of the questions completed were excluded,
	Questionnaires submitted with an atypical timestamp	None identified,
	Statistical correction	No.
